# Chloroquine Restores eNOS Signaling in Shunt Endothelial Cells via Inhibiting eNOS Uncoupling

**DOI:** 10.3390/ijms26031352

**Published:** 2025-02-05

**Authors:** Ying Liang, Wojciech Ornatowski, Qing Lu, Xutong Sun, Manivannan Yegambaram, Anlin Feng, Yishu Dong, Saurabh Aggarwal, Hoshang J. Unwalla, Jeffrey R. Fineman, Stephen M. Black, Ting Wang

**Affiliations:** 1 Center for Translational Science, Florida International University, Port Saint Lucie, FL 34987, USA; 2 Department of Environmental Health Sciences, Florida International University, Miami, FL 33199, USA; 3 Department of Cellular and Molecular Medicine, Florida International University, Miami, FL 33199, USA; 4 Department of Pediatrics, University of California, San Francisco, CA 94143, USA; 5 Cardiovascular Research Institute, University of California, San Francisco, CA 94158, USA

**Keywords:** CHD, chloroquine, nitric oxide, PAH

## Abstract

Pulmonary arterial hypertension (PAH) is characterized by increased lung vascular stiffness and impaired vessel relaxation, primarily due to reduced nitric oxide (NO) production in endothelial cells. Recent studies indicate that chloroquine, an autophagy inhibitor, may help lower pulmonary arterial pressure and enhance lung vascular function. This study investigates the mechanisms underlying the chloroquine-mediated restoration of NO bioavailability in endothelial cells derived from aortopulmonary shunt lambs, a relevant model for congenital heart defect (CHD)-associated PAH. We found that NO production was significantly reduced in shunt pulmonary artery endothelial cells (PAECs), attributable to decreased levels of tetrahydrobiopterin (BH_4_) and diminished expression of GTP cyclohydrolase 1 (GCH1), despite a slight increase in endothelial nitric oxide synthase (eNOS) levels. Chloroquine robustly restored endothelial NO production, which correlated with increased BH_4_ levels and restored GCH1 expression. The mechanistically upregulated carboxyl terminus of Hsp70-interacting protein (CHIP) in shunt PAECs is responsible for heightened GCH1 degradation, and chloroquine disrupted the assembly of the GCH1-HSP70-CHIP complex to preserve cellular GCH1. Similarly, another autophagy inhibitor, bafilomycin A1, demonstrated comparable effects. These findings suggest that autophagy inhibition can effectively enhance NO synthesis in endothelial cells experiencing depleted NO bioavailability, presenting a potential therapeutic strategy for managing PAH.

## 1. Introduction

Pulmonary arterial hypertension (PAH) is characterized by pulmonary endothelium remodeling and loss of vessel reactivity, processes that are particularly pronounced in patients with congenital heart defects (CHDs) and increased pulmonary blood flow (PBF) [1]. CHDs, the most common birth defect in the United States, affect nearly 40,000 infants annually, with about 25% classified as critical CHDs requiring early surgical intervention [2]. A large sub-set of these defects expose the pulmonary circulation to elevated blood flow and pressure, leading to endothelial remodeling, reduced vascular cross-sectional area, and severe complications such as hypoxia, heart failure, and death [3]. While surgical repair improves survival, residual vascular reactivity and remodeling contribute to significant long-term morbidity, including pulmonary hypertension and right heart failure [3]. The healthcare burden of CHD-associated PAH is substantial, exceeding USD 6 billion annually in direct medical costs [4]. The chronic nature of these conditions imposes further challenges, underscoring the urgent need for therapies targeting the molecular mechanisms underlying pulmonary vascular reactivity and remodeling.

Recent studies suggest that chloroquine, an antimalarial drug [5], may serve as a promising therapeutic strategy for PAH, improving vascular reactivity and protecting the pulmonary vasculature. Experimental evidence indicates that chloroquine exerts significant beneficial effects in PAH though multiple mechanisms [6,7]. It acts as a potent vasodilator, reducing pulmonary vascular resistance by blocking voltage-dependent calcium channels and inhibiting store-operated and receptor-operated calcium channels in pulmonary artery smooth muscle cells [7]. Additionally, chloroquine may activate large-conductance calcium-activated potassium (BK) channels [8], contributing to membrane hyperpolarization and improved vessel reactivity. Chloroquine has also been shown to prevent the development of PAH, reduce right ventricular hypertrophy, attenuate pulmonary artery remodeling, and slow the progression of established PAH in experimental models [6]. At the cellular level, it inhibits autophagy pathways, increases BMPR-II protein levels by preventing its lysosomal degradation, and reduces PASMC proliferation while promoting apoptosis [9]. These combined vasodilative, anti-proliferative, and anti-autophagic effects highlight chloroquine’s potential in mitigating pulmonary vascular dysfunction.

A central factor in the pathogenesis of CHD-PAH is the depletion of endothelial nitric oxide (NO), a critical vasodilator produced by endothelium to maintain pulmonary vascular tone [10,11,12]. Chloroquine has been reported to modulate NO synthesis in various systems [13,14,15,16] but has not yet been studied in the context of CHD-PAH. In healthy endothelium, NO is synthesized by endothelial nitric oxide synthase (eNOS) from L-arginine [17]. Once produced, NO diffuses into surrounding smooth muscle cells, activating soluble guanylate cyclase and raising cyclic GMP (cGMP) levels, leading to vasodilation [18]. In CHDs with increased PBF, reduced NO bioavailability exacerbates pulmonary vascular reactivity and drives vascular remodeling, a key contributor to disease progression [10,11,12]. Although chloroquine has shown promise in reducing vascular remodeling in adult PAH via improvement of calcium handling [7], its effects on CHD-PAH or the specific mechanisms of NO synthesis in this setting remain unknown. This gap in knowledge highlights the critical need for further investigation into how chloroquine modulates vessel reactivity and pulmonary vascular health in CHD-PAH.

In this study, we aim to address this gap by investigating the molecular pathways though which chloroquine enhances NO production in pulmonary endothelial cells from our ovine model of CHD with increased PBF (shunt). By exploring the interplay between autophagy, eNOS activity, and NO bioavailability, we seek to uncover chloroquine’s potential to reverse endothelial dysfunction and attenuate pulmonary vascular remodeling. Understanding these mechanisms will provide novel insights into targeting pulmonary vascular injury and improving outcomes for patients with CHD-related pulmonary arterial hypertension.

## 2. Results

### 2.1. Chloroquine Restores Reduced NO in Shunt Pulmonary Artery Endothelial Cells (PAECs)

**Figure 1:**
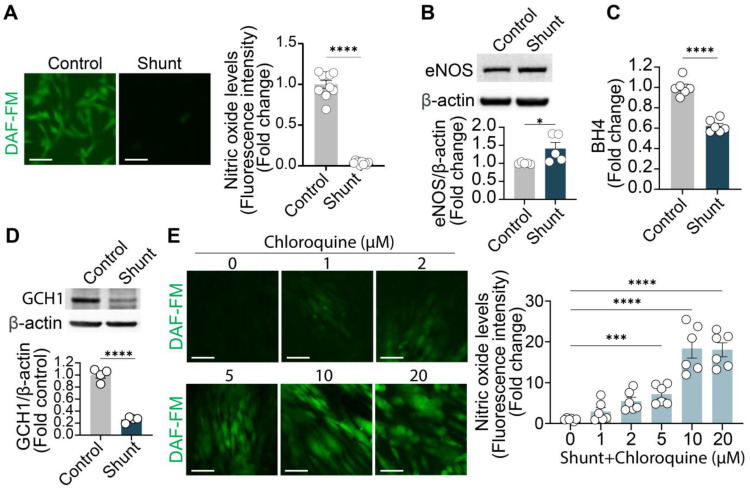
Chloroquine restores NO production in shunt PAECs. (**A**) (Left) PAECs from control and shunt lambs are stained with the fluorescent NO probe DAF-FM. Scale bar = 50 μm. (Right) Quantification of the DAF-FM signal. (**B**) Western blot analysis shows a slightly increased eNOS protein level in shunt PAECs. (**C**) BH_4_ levels are significantly lower in shunt PAECs compared to control PAECs. (**D**) GCH1 protein expression is downregulated in shunt PAECs. (**E**) Chloroquine increases NO production in shunt PAECs in a dose-dependent manner (2–10 μM, 24 h). Scale bar = 50 μm. All experiments were performed with at least three biological replicates, and each replicate is indicated as a single dot. * *p* < 0.5, between the two groups; ***, *p* < 0.001; ****, *p* < 0.0001.

Aortopulmonary shunts lead to postnatal pulmonary hypertension, increased pulmonary blood flow, and vascular remodeling [19]. Reduced nitric oxide bioavailability is a key contributor of impaired vessel dilation [20]. To evaluate NO production in the shunt model, PAECs from control and shunt lambs were stained with the fluorescent NO probe DAF-FM. Shunt ECs displayed significantly reduced NO production compared to control ECs (Figure 1A).

eNOS is the primary enzyme responsible for NO synthesis in the vascular endothelium [21]. Contrary to reduced NO bioavailability, Western blot analysis revealed that eNOS expression was slightly increased, rather than decreased, in shunt ECs compared to controls (Figure 1B). The slightly elevated eNOS levels in shunt ECs produce less NO, indicating a decrease in eNOS efficiency [22]. Tetrahydrobiopterin (BH_4_) is an essential cofactor for NO production by NOS enzymes [23]. BH_4_ levels were measured in control and shunt ECs, revealing that shunt ECs exhibited significantly lower BH_4_ levels compared to controls (Figure 1C). This reduction in BH_4_ was accompanied by a decrease in GTP Cyclohydrolase 1 (GCH1, Figure 1D), the rate-limiting enzyme in BH_4_ biosynthesis [24]. These findings suggest that the diminished NO production in shunt ECs is associated with the downregulation of GCH1 and a corresponding reduction in BH4 levels.

**Figure 2:**
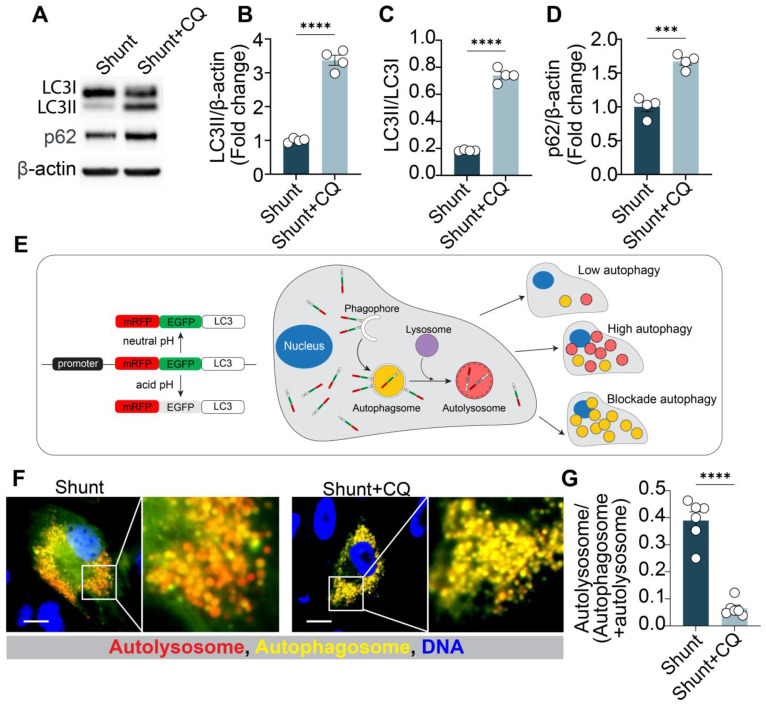
Chloroquine exerts strong autophagic inhibition in shunt PAECs. (**A**–**D**) Chloroquine (10 μM, 24 h) increases LC3II levels, LC3II/LC3I ratio, and p62 protein levels in shunt PAECs. (**E**) Autophagic flux is visualized using the mRFP-GFP-LC3 plasmid [26], which expresses LC3 fusion protein with both monomeric red fluorescent protein (mRFP) and green fluorescent protein (GFP). In neutral pH conditions (autophagosome), fusion protein exhibits yellow color (red + green). In acidic environments (autolysosome), GFP fluorescence is quenched and fusion protein exhibits red color. Levels of autophagic activity can be visualized and compared. (**F**) PAECs pre-transfected with mRFP-GFP-LC3 were treated with 10 μM chloroquine for 24 h, and LC3 puncta were detected via fluorescence microscopy. Scale bar = 20 μm. (**G**) Ratio of autolysosome (red)/(autolysosome + autophagosome [yellow]) is quantified to indicate autophagic flux activity. All experiments were performed with at least three biological replicates, and each replicate is indicated as a single dot. ***, *p* < 0.001 between the two groups; ****, *p* < 0.0001.

Chloroquine or CQ, an antimalarial drug known to inhibit autophagy, has been reported to restore NO levels [15]. We applied chloroquine treatment to shunt ECs and observed a significant, dose-dependent restoration in NO levels (1–20 μM) (Figure 1E). Chloroquine treatment did not further increase NO levels in control ECs (Supplementary Figure S1). Considering the side effects of chloroquine treatment (Supplementary Figure S1), 10 μM chloroquine was chosen for following experiments. At the same time, chloroquine’s effects on autophagic flux blockade was also validated in shunt ECs (Figure 2). Chloroquine treatment (10 μM, 24 h) increased LC3II, LC3II/LC3I ratio, and p62 protein levels (Figure 2A–D). This combination suggested a paused autophagic flux with accumulation of all essential proteins [25]. To further monitor autophagy, we used the mRFP-GFP-LC3 plasmid [26], which expresses LC3 fused with both monomeric red fluorescent protein (mRFP) and green fluorescent protein (GFP). In acidic environments (autolysosome), GFP fluorescence is quenched, while only RFP fluorescence remains stable. An increase in yellow puncta (both GFP- and RFP-positive) reflects autophagosome accumulation, whereas red-only puncta (GFP-negative/RFP-positive) indicate successful fusion with lysosomes (Figure 2E). Fluorescence microscopy showed that chloroquine inhibited autophagy by blocking the fusion of late endosomes with lysosomes (Figure 2F,G), consistent with previous reports. These findings suggest that chloroquine disrupted autophagy in shunt ECs.

### 2.2. Chloroquine Restores NO Levels in Shunt ECs by Upregulating GCH1 Protein Expression

**Figure 3:**
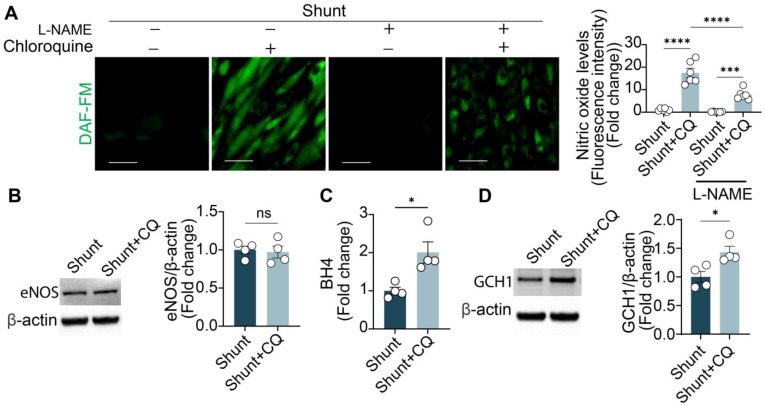
Chloroquine increases NO by upregulating GCH1 protein levels in shunt PAECs. (**A**) (Left) Shunt PAECs are pre-treated with L-NAME (a nonselective NOS inhibitor, 1 mM, 30 min) and then treated with chloroquine (10 μM, 24 h). Cells are then stained with the fluorescent NO probe DAF-FM. Scale bar = 50 μm. (Right) Quantification of the DAF-FM signal. (**B**) Western blot analysis reveals no significant change in eNOS protein levels in shunt PAECs treated with chloroquine (10 μM, 24 h) compared to untreated shunt PAECs. (**C**) BH_4_ levels are significantly increased in shunt PAECs treated with chloroquine (10 μM, 24 h) compared to untreated shunt PAECs. (**D**) GCH1 protein expression is upregulated in shunt PAECs following treatment with chloroquine (10 μM, 24 h). All experiments were performed with at least three biological replicates, and each replicate is indicated as a single dot. *, *p* < 0.05 between two groups; ***, *p* < 0.001; ****, *p* < 0.0001; ns, not significant.

To further investigate chloroquine’s effect on NO production, PAECs from shunt lambs were pre-treated with L-NAME (1 mM), a nonselective NOS inhibitor, for 30 min, followed by stimulation with 10 μM chloroquine for 24 h. Chloroquine significantly restored NO levels in shunt ECs, but this increase was abolished by L-NAME treatment (Figure 3A), indicating that chloroquine-induced NO production is mediated by NOS activity. Western blot analysis confirmed no significant change in eNOS protein levels in shunt ECs after chloroquine treatment (Figure 3B), suggesting that the increase in NO production was not due to an upregulation of eNOS protein. Notably, chloroquine treatment (10 μM, 24 h) significantly elevated BH_4_ levels in shunt ECs compared to untreated cells (Figure 3C). This increase in BH_4_ was accompanied by a significant upregulation of GCH1 protein expression (Figure 3D). In contrast, chloroquine did not further elevate BH4 levels or GCH1 protein expression in control PAECs (Supplementary Figure S2). These results suggest that chloroquine enhances NO production in shunt ECs by promoting GCH1 expression and restoring BH_4_ levels, independent of any changes in eNOS protein levels.

### 2.3. Chloroquine Upregulates GCH1 by Inhibiting Protein Degradation

**Figure 4:**
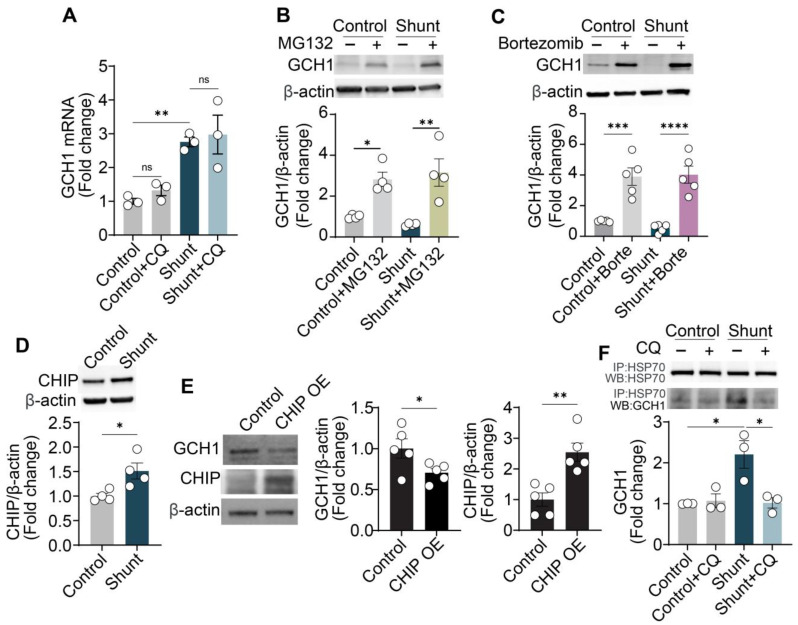
Chloroquine upregulates GCH1 by inhibiting its degradation. (**A**) RT-PCR shows GCH1 mRNA levels are increased in shunt PAECs, while chloroquine (10 μM, 24 h) exposure does not alter GCH1 mRNA levels in either control or shunt PAECs. (**B**) MG132 (10 μM, 24 h) significantly increases GCH1 protein levels in both control and shunt PAECs. (**C**) Bortezomib (10 nM, 24 h) significantly increases GCH1 protein levels in both control and shunt PAECs. (**D**) CHIP levels are elevated in shunt PAECs. (**E**) Overexpression of CHIP (CHIP OE) via DNA plasmid transduction in control PAECs significantly reduces GCH1 protein levels. (**F**) Co-immunoprecipitation demonstrates enhanced binding of HSP70 and GCH1 in shunt PAECs, which is reduced following treatment with chloroquine (10 μM, 24 h). All experiments were performed with at least three biological replicates, and each replicate is indicated as a single dot. *, *p* < 0.05 between the two groups; **, *p* < 0.01; ***, *p* < 0.001; ****, *p* < 0.0001; ns, not significant.

We next investigated the mechanism though which chloroquine upregulates GCH1 in shunt ECs. Contradictory to reduced protein expression, GCH1 mRNA levels were elevated in shunt ECs compared to controls (Figure 4A). And chloroquine treatment (10 μM, 24 h) had no additional effect on GCH1 mRNA levels in either group (Figure 4A). This suggests that chloroquine regulates GCH1 expression post-transcriptionally.

MG132 (Figure 4B), a well-known proteasome inhibitor, and Bortezomib (Figure 4C), an FDA-approved proteasome inhibitor for the treatment of multiple myeloma [27], significantly increased GCH1 protein levels in both control and shunt ECs, indicating that GCH1 undergoes rapid and regulatable proteasomal degradation. Additionally, CHIP (C-terminal Hsp70-interacting protein), a known E3 ubiquitin ligase involved in protein degradation [28], was found to be upregulated in shunt ECs (Figure 4D). Overexpression of CHIP with a DNA plasmid (CHIP-OE) significantly reduced GCH1 protein levels in control ECs (Figure 4E), highlighting its role in GCH1 degradation.

HSP70 is the active molecular chaperon for CHIP in shunt PAECs [29], and we have shown that GCH1-HSP70 interaction is the key step for CHIP-mediated GCH1 ubiquitination and degradation [30]. We next confirmed the effects of chloroquine on GCH1-HSP70 interaction in shunt PAECs. Co-immunoprecipitation experiments revealed increased binding between GCH1 and HSP70 in shunt ECs, which facilitates GCH1 degradation (Figure 4F). Interestingly, chloroquine treatment (10 μM, 24 h) reduced the interaction between HSP70 and GCH1, providing a possible mechanism for how chloroquine stabilizes GCH1 protein levels.

### 2.4. Bafilomycin A1 Mimics the Effect of Chloroquine on NO Production in Shunt ECs

**Figure 5:**
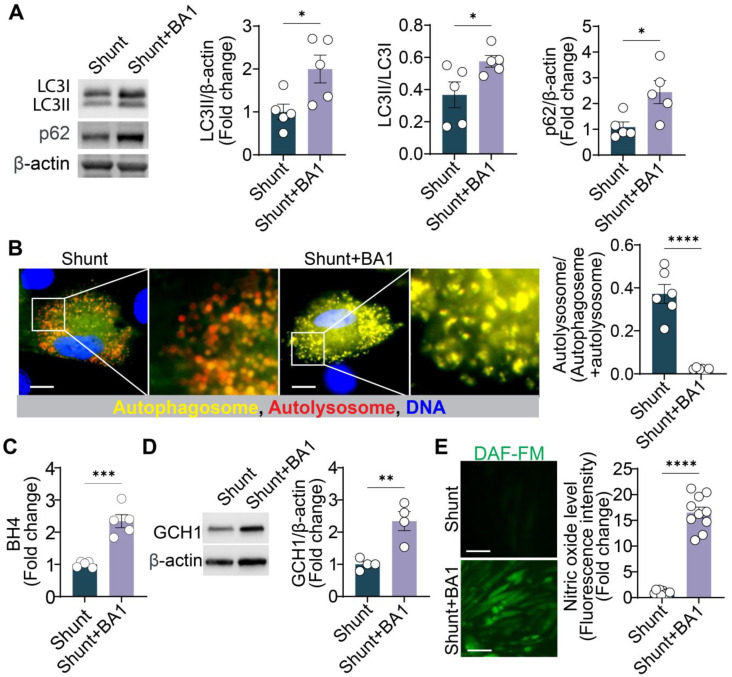
Bafilomycin A1, another autophagy inhibitor, mimics the effect of chloroquine on NO production in shunt PAECs. (**A**) Bafilomycin A1 (50 nM, 24 h) increases LC3II levels, LC3II/LC3I ratio, and p62 protein levels in shunt PAECs, indicating inhibited autophagic flux. (**B**) Shunt PAECs pre-transfected with mRFP-GFP-LC3 are treated with Bafilomycin A1 (50 nM, 24 h), and LC3 puncta are detected via fluorescence microscopy. Scale bar = 20 μm. The ratio of autolysosome (red)/(autolysosome + autophagosome [yellow]) is quantified to indicate autophagic flux activity. (**C**) BH_4_ levels are significantly increased in shunt PAECs treated with Bafilomycin A1 (50 nM, 24 h). (**D**) GCH1 protein expression is upregulated in shunt PAECs treated with Bafilomycin A1 (50 nM, 24 h). (**E**) Bafilomycin A1 (50 nM, 24 h) increases NO production in shunt PAECs. Scale bar = 50 μm. All experiments were performed with at least three biological replicates, and each replicate is indicated as a single dot. *, *p* < 0.05 between the two groups; **, *p* < 0.01; ***, *p* < 0.001; ****, *p* < 0.0001.

Given that chloroquine inhibits autophagy, we investigated whether another autophagy inhibitor, bafilomycin A1, could produce similar effects in shunt ECs. Bafilomycin A1 (50 nM, 24 h) increased the LC3II levels, LC3II/LC3I ratio, and p62 protein levels in shunt ECs, confirming that autophagic flux was inhibited (Figure 5A). Fluorescence microscopy using the mRFP-GFP-LC3 plasmid further demonstrated increased LC3 yellow puncta (accumulated autophagosome) after treatment with bafilomycin A1, similar to the effects observed with chloroquine (Figure 5B).

Bafilomycin A1 treatment (50 nM, 24 h) also restored BH_4_ levels in shunt ECs, which were significantly increased compared to untreated cells (Figure 5C). This increase in BH_4_ was accompanied by upregulation of GCH1 protein expression (Figure 5D). Finally, bafilomycin A1 treatment enhanced NO production in shunt ECs (Figure 5E), mirroring the effect of chloroquine. In contrast, Bafilomycin A1 treatment only slightly increased GCH1 protein levels, followed by an increase in BH_4_ levels and NO production, in control ECs (Supplementary Figure S3). These findings suggest that the inhibition of autophagy is a key mechanism by which both chloroquine and bafilomycin A1 restore NO production in shunt ECs.

### 2.5. Result Summary

In summary, NO production is reduced in shunt ECs due to the downregulation of GCH1 and diminished BH_4_ levels. Chloroquine treatment restores NO production by upregulating GCH1 protein levels though the inhibition of its degradation, potentially by disrupting the interaction between HSP70 and GCH1. These effects are associated with the inhibition of autophagic flux. Notably, another autophagy inhibitor, bafilomycin A1, produces similar effects on NO production and GCH1 expression in shunt ECs, further supporting the role of autophagy in regulating NO production.

## 3. Discussion

This study investigates the mechanisms behind reduced NO production in PAECs from aortopulmonary shunt lambs, an established model for CHD-PAH [19]. This study highlights the critical role of autophagy modulation in improving endothelial NO bioavailability, offering a promising therapeutic avenue for PAH and related vascular diseases. By identifying autophagic flux as a key regulator of NO production, this research establishes a novel target for intervention. It underscores the therapeutic potential of restoring BH_4_ levels though upregulation of GCH1, supporting endothelial function and enhancing NO synthesis. Importantly, these findings extend beyond PAH, suggesting broader applications in managing vascular diseases characterized by inflammation, remodeling, and thrombosis.

NO is essential for maintaining pulmonary vascular homeostasis, playing a critical role in regulating vascular tone, inhibiting vascular remodeling, controlling inflammation, and preventing thrombosis [17]. As a potent vasodilator produced by eNOS in vascular systems, NO leads to relaxing smooth muscle cells, reducing pulmonary arterial pressure, and preventing excessive vasoconstriction. In pulmonary vascular diseases like CHD-PAH, reduced NO production leads to increased vasoconstriction, elevated pulmonary pressure, and impaired blood flow, driving disease progression [31]. Additionally, NO inhibits the proliferation and migration of smooth muscle cells and fibroblasts [32], which are involved in pathological vascular remodeling—a hallmark of PAH that results in the thickening of blood vessel walls and increased resistance to blood flow. Furthermore, NO’s anti-inflammatory properties reduce endothelial dysfunction, a critical factor in pulmonary vascular diseases where inflammation exacerbates vascular damage [33]. NO also maintains an antithrombotic environment by inhibiting platelet aggregation and adhesion, and reduced NO levels increase the risk of thrombosis, further contributing to vascular occlusion and disease progression [34]. NO plays a central role in vascular homeostasis, and this current study demonstrates that the regulation of NO synthesis through therapies such as chloroquine can successfully restore NO levels by inhibiting autophagic flux. It provides a possible solution to counteract the pathological effects of reduced NO production in PAH.

Autophagy is known to play a very complex role in pulmonary vasculature in PAH. The relationship between autophagy and endothelial cell behavior in PAH is intricate, as autophagy can influence the transition of endothelial cells from an initial apoptotic state to a later hyper-proliferative state [35]. In some instances, inhibiting autophagy has been shown to increase apoptosis and reduce the proliferation of endothelial cells, while stimulating autophagy can yield the opposite effects [36]. Both activation [37] and inhibition [38] of autophagy in pulmonary vascular cells have demonstrated potential in alleviating pulmonary hypertension, depending on the specific context. Understanding the precise timing and mechanisms of autophagy regulation is essential for developing new targeted therapies for PH. Independent of pharmacological intervention, autophagic activity fluctuates throughout the disease’s progression, serving both protective and potentially detrimental roles depending on the stage and specific cellular context. This underscores the necessity for further research to fully elucidate autophagy’s role and potentially harness its therapeutic benefits in pulmonary hypertension.

In this study, we demonstrate that inhibiting autophagy enhances NO production in the lung vascular endothelium of aortopulmonary shunt lambs, an established model of CHD-associated pulmonary arterial hypertension. The data highlight the critical relationship between autophagy, ATP availability, and HSP70 activity in maintaining GCH1 homeostasis, which is essential for sufficient NO synthesis. In shunt ECs, decreased NO production correlates with reduced BH_4_ levels and impaired GCH1 protein stability [30], despite a slight upregulation in eNOS expression, which is considered as a response of negative feedback. By inhibiting autophagic flux with agents such as chloroquine and bafilomycin A1, we observed an upregulation of GCH1 protein levels, increased BH_4_ synthesis, and a subsequent restoration of NO availability. This autophagy inhibition seems to interrupt HSP70-GCH1 interaction, which protects GCH1 from proteasomal degradation. We believe autophagy intervention in this particular shunt condition favors HSP70, a known autophagy-relevant factor [39], to override its major cellular competitive chaperone HSP90 [40], over GCH1 binding and regulation. These cellular events underscore the findings of our current study, suggesting that autophagy inhibition can strategically increase NO bioavailability by sustaining GCH1 homeostasis, providing a potentially valuable therapeutic strategy to counteract the endothelial dysfunction characteristic of PAH and other vascular diseases where NO production is compromised. As a weakness and also the future direction of the current study, we will examine the effects of chloroquine in aortopulmonary shunt lambs with in vivo settings to examine the therapeutic efficacy of chloroquine in this devastating disease.

Chloroquine (or other autophagy modulators) may offer benefits for pulmonary hypertension but requires cautious use due to potential cardiovascular risks. It acts as a potent pulmonary vasodilator, reducing hypoxia-induced pulmonary hypertension, protects against right heart remodeling in PAH [7], and improves quality of life while slowing disease progression. However, it can prolong the QT interval, increasing the risk of ventricular arrhythmias, and may cause severe cardiotoxicity, including cardiomyopathy, with higher risks in patients with pre-existing cardiac conditions. Additionally, studies in hypertensive rats have shown increased mortality, QTc prolongation, and myocardial damage, raising concerns for hypertensive patients. While chloroquine shows promise, its use must be carefully monitored, particularly in those with cardiovascular disease or hypertension. As a safer alternative, BH4 supplementation with its precursors, such as sepiapterin [30], may provide an effective therapy with fewer potential side effects.

In the context of our study, autophagy inhibition appears to cause a temporary reduction in ATP levels [41]. This ATP depletion could serve as a cellular signal to disassemble the HSP70-CHIP complex [42], potentially stabilizing critical proteins like GCH1. Under normal conditions, HSP70, in partnership with CHIP (an E3 ubiquitin ligase), targets specific proteins for degradation, which may contribute to lower GCH1 protein levels and thus reduce NO production in CHD-PAH [30]. However, upon autophagy inhibition and subsequent ATP depletion, HSP70-CHIP disassembly likely delays GCH1 degradation, providing a transient boost in NO synthesis. This could act as a protective mechanism, allowing the cell to rapidly adapt to the altered metabolic and stress conditions resulting from autophagy loss. From an interpretive perspective, cells might deploy this mechanism to prevent excessive NO loss when autophagy is inhibited, safeguarding critical vascular functions reliant on NO availability. The ATP-dependent nature of HSP70-CHIP function suggests that in low-energy conditions, such as those following autophagy inhibition, cells can reprioritize ATP for other essential processes, thereby inhibiting GCH1 degradation. This could explain why both chloroquine and bafilomycin A1, by inhibiting autophagy and inducing transient ATP depletion, restore NO production by maintaining GCH1 levels. Therefore, the findings propose that autophagy inhibition may not only help stabilize NO production but also activate broader adaptive responses that enhance cell resilience against conditions associated with endothelial dysfunction, such as PAH.

## 4. Materials and Methods

### 4.1. Chemical and Biochemical Reagents

Chloroquine phosphate (CQ) was purchased from Cayman Chemical (Ann Arbor, MI, USA). NG-Nitro-L-arginine Methyl Ester (L-NAME) was purchased from Sigma-Aldrich (Burlington, MA, USA). Bafilomycin A1(BA-1) was acquired from MedchemExpress (Monmouth Junction, NJ, USA). DAF-FM diacetate, DMEM culture media, protein BCA assay, and RIPA lysis buffer were purchased from Thermo-Fisher (Waltham, MA, USA). GCH1 antibody was purchased from Proteintech (Rosemont, IL, USA). eNOS antibody was purchased from BD Biosciences (San Jose, CA, USA). LC3, p62, β-actin, and CHIP antibodies were purchased from Cell Signaling (Danvers, MA, USA). All other chemicals were purchased from Sigma-Aldrich if not else stated.

### 4.2. Pulmonary Artery Endothelial Cells (PAECs)

An anastomosis between the ascending aorta and main pulmonary artery was created in anesthetized fetal lambs to form the shunt model [19]. Four weeks after spontaneous delivery, lung tissues were harvested for pulmonary artery endothelial cell isolation using an established protocol [43]. PAECs were then cultured in full DMEM culture media containing glucose, sodium pyruvate, glutamine, 10% fetal bovine serum (Lonza, Cambridge, MA, USA), and antibiotics (Penicillin and Streptomycin), at 37 °C with 5% CO_2_. PAECs were passaged upon reaching 95% confluence, with experiments performed on cells between passages 7 and 9, ensuring passage consistency. All protocols were approved by the Committee on Animal Research of the University of California, San Francisco [19].

### 4.3. Cellular Nitric Oxide Measurement

NO levels in cultured PAECs were determined by using DAF-FM diacetate, a cell-permeable fluorescent dye, according to the manufacture’s protocol. Briefly, PAECs were rinsed with PBS post treatment and incubated in basal DMEM media with DAF-FM diacetate (5 μM) for 30 min in the dark at 37 °C, then subjected to fluorescence microscopy [43]. Fluorescent images were obtained by using a KEYENCE BZ-X810 digital microscope. Relative fluorescent intensities were analyzed by using ImageJ 3 software version 1.53 to represent relative cellular NO levels.

### 4.4. Quantification of BH_4_ Levels by ELISA

Post treatment, PAECs were gently washed with cold PBS and dissociated using trypsin. Cells were then centrifuged for 5 min at 1000× *g* for pellet collection and washed further with cold PBS. Cells were then re-suspended with PBS and lysed by a repeated freeze–thaw process. Cell extraction was performed after centrifugation (10 min, 1500 g, 4 °C) for the measurement of total BH_4_ levels. The assay was performed using a commercially available BH_4_ ELISA kit (Novus Biologicals, Centennial, CO, USA). The absorbance was measured using a plate reader (BioTek, Winooski, VT, USA) at 405 nm.

### 4.5. Western Blotting

A standard Western blotting protocol was utilized [44]. Briefly, after treatment, PAECs were washed with cold phosphate-buffered saline (PBS), harvested, and homogenized in RIPA lysis buffer containing protease and phosphatase inhibitor cocktails (Sigma-Aldrich). The cell protein lysates were centrifuged at 13,000× *g* for 15 min at 4 °C, and the supernatant was collected. The protein samples were then boiled with Laemmli Sample Buffer (Bio-Rad, Hercules, CA, USA) and β-Mercaptoethanol. Electrophoresis was then performed for these protein samples (10 μg per lane) on 4–20% gradient SDS-PAGE gels (Thermo-Fisher) and transferred to PVDF membranes (Thermo-Fisher). Membranes were blocked with 5% BSA in Tris-buffered saline with Tween 20 (TBST, Thermo-Fisher) for 1 h and incubated overnight at 4 °C with primary antibodies diluted in the blocking buffer (with suggested dilution by manufacture). After washing with TBST three times, the membranes were incubated with HP-conjugated secondary antibodies (Thermo-Fisher) for 1 h at room temperature. Membranes were then washed with TBST three times to be incubated with a chemiluminescence reagent (West-Femto, Pierce, Rockford, IL, USA). Protein bands were visualized, recorded, and quantified with an iBright Imaging System (Thermo-Fisher).

### 4.6. Immunoprecipitation

After treatment, PAECs were washed with cold PBS and harvested for lysis with Pierce IP Lysis Buffer (Thermo-Fisher) with a protease cocktail (Thermo-Fisher). Protein lysates were centrifuged at 13,000× *g* for 15 min at 4 °C and the supernatant was collected. HSP70 antibody (5 μg/mL) was incubated with protein lysates overnight in 4 °C. Then, Pierce Protein A/G Plus Agarose beads (40 μL, Thermo-Fisher) were added to the lysates and incubated with gentle rotation for 3 h. The beads were then washed with IP Lysis Buffer 3 times and boiled in Laemmli Sample Buffer for 10 min. These boiled samples were further analyzed by Western blotting.

### 4.7. mRFP-GFP-LC3 Puncta Analysis

PAECs seeded in confocal dishes (VWR, Radnor, PA, USA) were transfected with the plasmid (Addgene, Watertown, MA, USA) encoding fluorescent mRFP-GFP-LC3 fusion protein for 24 h. The cells were randomly divided into different groups for different treatments. After the treatment, cells were stained with DAPI at room temperature for 10 min, and live cell fluorescent images were taken using the KEYENCE BZ-X810 digital microscope.

### 4.8. RNA Isolation, Reverse Transcription, and Quantitative Real-Time PCR

Total RNA was extracted from endothelial cells using RNeasy Kits (Qiagen, Venlo, The Netherlands) according to the manufacturer’s instructions. The quantity of RNA was measured via a Nanophotometer (Thermo-Fisher). Reverse transcription was performed using the QuantiTect Reverse Transcription Kit (Qiagen), and quantitative real-time PCR (qPCR) analysis was performed with TaqMa Gene Expression Master Mix and Taqman Gene Expression assay (Thermo-Fisher) using a QuantStudio Real-Time PCR System (Thermo-Fisher). Relative abundance of each target transcript was normalized to GAPDH, calculated using the 2^−ΔΔCt^ method.

### 4.9. Statistical Analysis

GraphPad Prism 9.5.0 was used to conduct statistical analysis in this study. Mean ± SEM was calculated in all experiments, while statistical significance was determined by Student’s *t*-test with two groups or an ANOVA test with multiple comparisons. *p* < 0.05 is considered statistically significant.

## 5. Conclusions

Taken together, this study confirms that chloroquine restores NO levels in CHD-PAH by upregulating GCH1 expression and increasing BH_4_ availability though inhibition of autophagy and protein degradation. Our findings demonstrate that chloroquine treatment reverses endothelial dysfunction by preventing GCH1 degradation via suppression of CHIP-mediated ubiquitination, leading to improved NO synthesis despite unchanged eNOS protein levels. These results suggest that targeting autophagy and proteasomal degradation pathways may provide a novel therapeutic strategy for CHD-PAH.

## Data Availability

All original research data supporting the reported results can be made available upon request.

## Supplementary Materials

The following supporting information can be downloaded at: https://www.mdpi.com/article/10.3390/ijms26031352/s1.
