# Supplementary material for: Chloroquine Restores eNOS Signaling in Shunt Endothelial Cells via Inhibiting eNOS Uncoupling
Source: Int J Mol Sci. 2025 Feb 5;26(3):1352. doi: 10.3390/ijms26031352 (PMC11818845; doi:10.3390/ijms26031352)
Supplement: Supplementary file 1 [file ijms-26-01352-s001.zip › ijms-3379682-supplementary.pdf]

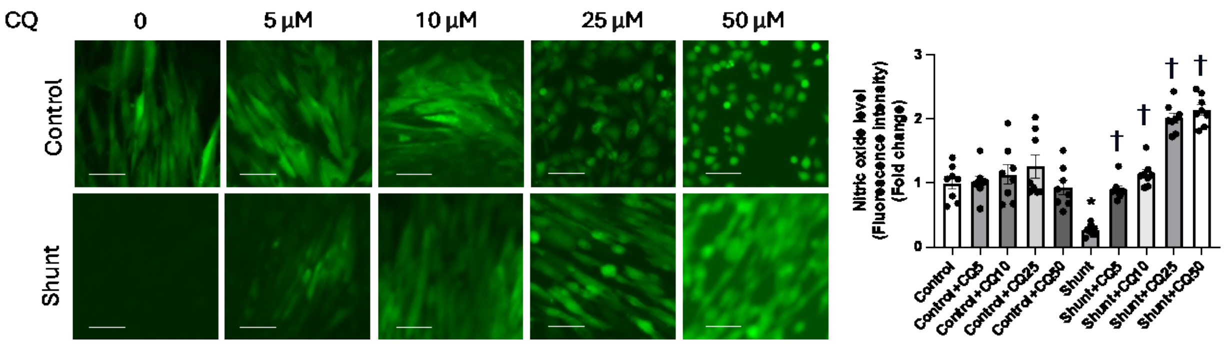

Supplementary Figure S1. Chloroquine increases NO production in shunt PAECs in a dose-dependent manner (5–50 μM, 24 hr) while not affect NO production in control PAECs. Control PAECs exhibit rounding morphological changes with high dose of chloroquine (25-50) μM. Shunt PAECs remained normal morphology with all concentrations of chloroquine treatment (5–50 μM). Scale bar = 50 μm. *, $p < 0.05$ compare with Control group; †, $p < 0.05$ compare with Shunt group.

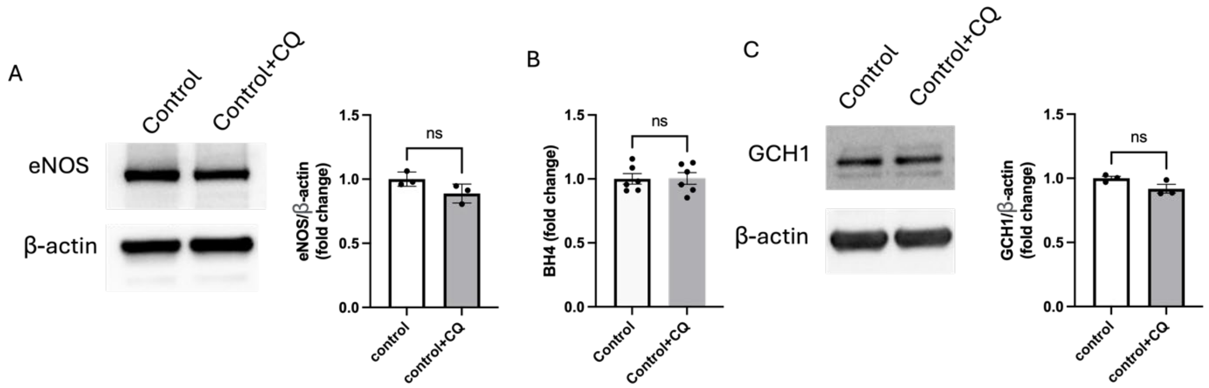

Supplementary Figure S2. Chloroquine does not affect GCH1 protein levels in control PAECs. (**A**) Western blot analysis shows no significant change in eNOS protein levels in control PAECs treated with chloroquine (10 μM, 24 h) compared to untreated control PAECs. (**B**) BH4 levels remain unaffected by chloroquine (10 μM, 24 h) in control PAECs. (**C**) GCH1 protein expression does not change in control PAECs following chloroquine treatment (10 μM, 24 h). All experiments were performed with at least three biological replicates. ns, not significant.

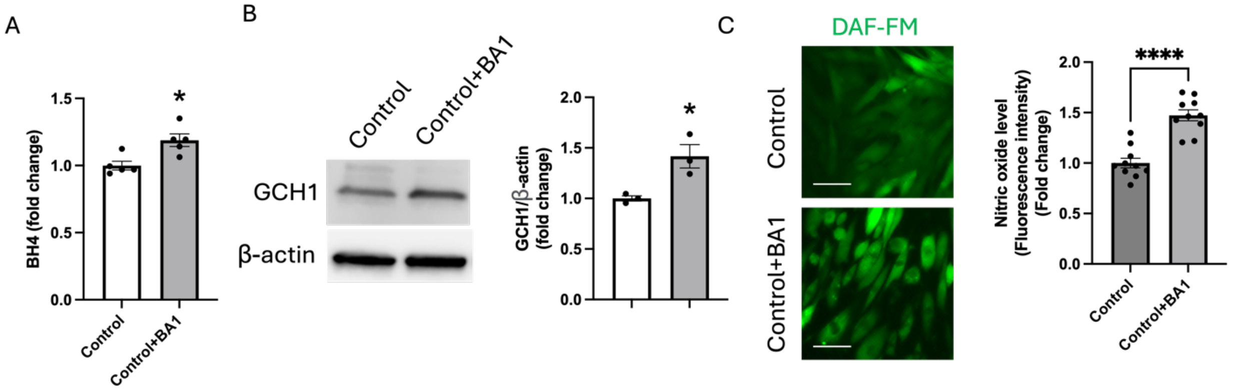

Supplementary Figure S3. Bafilomycin A1 slightly increases NO by upregulating GCH1 protein levels in control PAECs. (**A**) BH_4 levels are increased in control PAECs treated with Bafilomycin A1 (50 nM, 24 h). (**B**) GCH1 protein expression is upregulated in control PAECs treated with Bafilomycin A1 (50 nM, 24 h). (**C**) Bafilomycin A1 (50 nM, 24 h) increases NO production in control PAECs. Scale bar = 50 μm. All experiments were performed with at least three biological replicates. *, $p < 0.05$ between the two groups; ****, $p < 0.0001$.
